# Specificity, contexts, and reference groups matter when assessing autistic traits

**DOI:** 10.1371/journal.pone.0171931

**Published:** 2017-02-13

**Authors:** Morton Ann Gernsbacher, Jennifer L. Stevenson, Sebastian Dern

**Affiliations:** 1 Department of Psychology, University of Wisconsin–Madison, Madison, Wisconsin, United States of America; 2 Department of Psychology, Ursinus College, Collegeville, Pennsylvania, United States of America; 3 Faculty of Psychology and Neuroscience, Maastricht University, Maastricht, Netherlands; Nagoya Mental Clinic, JAPAN

## Abstract

Many of the personality and behavioral traits (e.g., social imperviousness, directness in conversation, lack of imagination, affinity for solitude, difficulty displaying emotions) that are known to be sensitive to context *(with whom*?*)* and reference group *(according to whom*?*)* also appear in questionnaire-based assessments of autistic traits. Therefore, two experiments investigated the effects of specifying contexts and reference groups when assessing autistic traits in autistic and non-autistic participants. Experiment 1 (124 autistic and 124 non-autistic participants) demonstrated that context matters when assessing autistic traits (*F*(1,244) = 267.5, *p* < .001, η^2^_p_ = .523). When the context of the Broad Autism Phenotype Questionnaire was specified as the participants’ out-group (e.g., “I like being around non-autistic people” or “I like being around autistic people”), both autistic and non-autistic participants self-reported having more autistic traits; when the context was specified as the participants’ in-group, participants reported having fewer autistic traits. Experiment 2 (82 autistic and 82 non-autistic participants) demonstrated that reference group matters when assessing autistic traits (*F*(2,160) = 94.38, *p* < .001, η^2^_p_ = .541). When the reference group on the Social Responsiveness Scale was specified as the participants’ out-group (e.g., “According to non-autistic people, I have unusual eye contact”), autistic participants reported having more autistic traits; when the reference group was their in-group, autistic participants reported having fewer autistic traits. Non-autistic participants appeared insensitive to reference group on the Social Responsiveness Scale. Exploratory analyses suggested that when neither the context nor the reference group is specified (for assessing autistic traits on the Autism-Spectrum Quotient), both autistic and non-autistic participants use the majority (“non-autistic people”) as the implied context and reference group.

## Introduction

The adage “context is everything” rings true when assessing personality and behavioral traits. Psychologists have known for decades that context can affect participants’ responses on personality and behavior questionnaires [1]. For example, in the 1940s, Eisenberg demonstrated that the item “Do you like to be alone?” elicits different answers if specified as “Do you like to be alone while working?” as opposed to “Do you like to be alone in a social setting?” [2]. Similarly, the item “Do you have difficulty speaking before a group?" elicits different answers if the group before one is speaking is specified as large versus small [3]. Context matters when assessing personality and behavior [4–6].

Psychologists have also known for decades that, in addition to context, reference group matters when assessing personality and behavior [7]. Leon Festinger [8] argued, over 60 years ago, that people can appraise personality and behavior only in reference to other people; indeed, Festinger argued that self- or other-appraisal is impossible without reliance on a reference group. Contemporary psychologists continue to demonstrate that participants’ responses on personality and behavioral questionnaires are greatly affected by the reference group with which the participants implicitly or explicitly compare themselves [9–12].

For example, men versus women rate themselves as less versus more caring and comforting to others when the reference group is women versus men [13]. Canadians versus Japanese report being more versus less socially impervious, odd or different, direct in their conversations, and concerned about their ability to take care of themselves and about the quality of their imagination when the reference group is Japanese versus Canadian [14]. Even university students who live in the Netherlands and are ethnically Chinese rate themselves as less likely to express their emotions when the reference group is ethnically Dutch students than when the reference group is other ethnically Chinese students [15]. Reference group matters when assessing personality and behavioral traits.

Because many of the traits that have been demonstrated to be sensitive to context and to reference group appear in autism assessments (e.g., social imperviousness, oddness or difference, directness in conversation, lack of imagination, reduced ability for self-care, affinity for solitude, difficulty displaying emotions), the experiments reported here examine the effects of context and reference group on the assessment of autistic traits. We use the identity-first terms “autistic traits” and “autistic participants” [16], rather than the person-first terms “autism-related traits” and “participants with autism” because identify-first language is not only recommended by psychologists [17] but also empirically demonstrated to be preferred by autistic people [18] and less prone to stigma [19]. And we predict that when assessing autistic traits, as when assessing other personality and behavioral traits, specific contexts and specific reference groups matter.

### Assessment of autistic traits

For diagnostic purposes, autistic traits are often assessed through interview and structured observation; for research purposes, autistic traits are often assessed via questionnaire. Our study focuses on assessing autistic traits through self-report questionnaires, although in our Discussion we will comment on the contrast between self-report and other-report questionnaires. Three of the most common self-report questionnaires are the Autism-Spectrum Quotient [20], the Broad Autism Phenotype Questionnaire [21], and the Social Responsiveness Scale [22].

#### The broad autism phenotype questionnaire

The Broad Autism Phenotype Questionnaire comprises 36 items, which are categorized into three inter-correlated subscales pertaining to social interaction, communication, and personality [21]. Although the Broad Autism Phenotype Questionnaire was created to assess autistic traits in non-autistic adults, most notably parents of autistic offspring, more recently it has been used to assess autistic traits in autistic adults [23,24] (but see [25]). According to the Broad Autism Phenotype Questionnaire’s authors, self-report, at least for non-autistic participants, generates scores similar to informant-report [26].

However, most of the Broad Autism Phenotype Questionnaire items resemble items that psychologists have demonstrated to be sensitive to context. For example, “I like being around other people,” “I enjoy chatting with people,” and “I would rather talk to people to get information than to socialize” are three items from the Broad Autism Phenotype Questionnaire’s social interaction subscale. But which “people” do respondents like to be around, enjoy chatting with, or prefer to solicit information from rather than interact socially with? Similarly, “I am ‘in tune’ with the other person during conversation” and “I feel disconnected or ‘out of sync’ in conversations with others” are two items from the Broad Autism Phenotype Questionnaire’s communication subscale. “People have to talk me into trying something new” and “People get frustrated by my unwillingness to bend” are two items from the personality subscale. But who are the “others” that respondents feel in-tune or out-of-sync with during conversation and which “people” have to push the respondent toward novelty or get frustrated by the respondent’s rigidity? Experiment 1 addresses these questions.

Experiment 1 manipulates the context of the items on the Broad Autism Phenotype Questionnaire to examine whether responses differ if “people” are specified as members of the respondents’ in-group versus out-group. By in-group, we mean a group in which respondents identify as members; by out-group, we mean a group in which respondents do not identify as members [27]. In-groups and out-groups are formed on the basis of racial identity, ethnic identity, and other identities, including disability identity [28] and autistic identity [29]. Because our study examines the effect of context when assessing autistic traits, we recruit to our study respondents who identify as autistic (i.e., “autistic people” are their in-group) and respondents who identify as non-autistic (i.e., “non-autistic people” are their in-group).

In-group membership facilitates social interaction [30], improves communication [31], and normalizes perceptions of extreme personality traits, such as rigidity or openness [32]. The Broad Autism Phenotype Questionnaire measures difficulty with social interaction, communication, and rigid personality. Therefore, we predict that specifying “people” as members of the respondents’ in-group will decrease the respondents’ self-reported difficulty interacting and communicating. Conversely, we predict that specifying “people” as members of the respondents’ out-group will increase the respondents’ self-reported difficulty interacting and communicating.

#### The social responsiveness scale

The Social Responsiveness Scale was created as a parent-report instrument for assessing autistic traits in children [33]. More recently, an adult version has been used as a self-report instrument for assessing autistic traits in both autistic and non-autistic adults [24, 34–36]. The Social Responsiveness Scale is a broad survey, which includes several items not typically included when assessing autistic traits, such as “I am not well coordinated (in physical activities),” “I have good self-confidence,” and “I have good personal hygiene.”

However, as with the items on the Broad Autism Phenotype Questionnaire, the items on the Social Responsiveness Scale are not specified. They lack grounding to a reference group. For items such as “I behave in ways that seem strange or bizarre” and “I’m regarded by others as odd or weird,” who are the others who consider the respondent weird and their behavior bizarre? When administering the Social Responsiveness Scale as a self-report instrument, some researchers tell respondents to respond in the way that “best describes how others would describe your behavior” ([34], p. 1649; [37], p. 463). But who are those others? Are they members of the respondents’ in-group or out-group? Does that matter? Experiment 2 answers these questions.

In Experiment 2, as in Experiment 1, half the respondents identify as autistic and half identify as non-autistic, enabling Experiment 2’s manipulation to also hinge on in- versus out-group membership. Experiment 2 manipulates whether items on the Social Responsiveness Scale reference the in-group (e.g., “According to autistic people, I behave in ways that seem strange or bizarre”), the out-group (e.g., “According to non-autistic people, I behave in ways that seem strange or bizarre”), or the respondents’ themselves (“I think that I behave in ways that seem strange or bizarre”). Out-group reference usually leads respondents to accentuate their own group-typical traits, while in-group reference leads respondents to attenuate their group-typical traits [13–15]. Therefore, we predict that respondents will accentuate their self-reported autistic traits when the out-group is referenced and will attenuate their autistic traits when the in-group is referenced.

#### The Autism-Spectrum Quotient

The Autism-Spectrum Quotient was created as a self-report questionnaire for both autistic and non-autistic adults [20]. It comprises 50 items, which are categorized into five inter-correlated subscales pertaining to social interaction, communication, attention to detail, attention switching, and imagination. However, low reliabilities have been reported for several of the subscales, and only two or three, rather than five, factors account for the item variability [38–40].

Many of the Autism-Spectrum Quotient items are unspecified with regard to context, similar to the Broad Autism Phenotype items investigated in Experiment 1. For example, the Autism-Spectrum Quotient items “I enjoy meeting new people” and “I prefer to do things with others rather than on my own” fail to specify which people the respondent enjoys meeting and prefers to do things with. Many of the Autism-Spectrum Quotient items are also unspecified with regard to reference group, similar to the Social Responsiveness Scale items investigated in Experiment 2. For example, the Autism-Spectrum Quotient items “I am a good diplomat” and “I tend to have very strong interests …” fail to specify the reference group for deeming oneself a good diplomat or the possessor of strong interests. In fact, some of the Autism-Spectrum Quotient items seem to demand a reference group, for example, “I tend to notice details that others do not” and “I often notice small sounds when others do not.”

In exploratory analyses, we estimate the context upon autistic and non-autistic participants implicitly rely when the context of an assessment of autistic traits is not specified; we correlate participants’ responses to Autism-Spectrum Quotient items that are unspecified with their responses to Broad Autism Phenotype items that are specified with an in- or out-group context. We also estimate the reference group that autistic and non-autistic participants implicitly consider if no reference group is specified for an assessment of autistic traits; we correlate participants’ responses to Autism-Spectrum Quotient items that are unspecified with their responses to Social Responsiveness items that are specified with an in-group reference, an out-group reference, or self-reference.

## Experiment 1

Experiment 1 examined the effect of context when assessing autistic traits by specifying “people” in the Broad Autism Phenotype Questionnaire items as either “autistic people” or “non-autistic people.” Half the participants in Experiment 1 identified as autistic, and half identified as non-autistic. Both autistic and non-autistic participants responded to both versions of the Broad Autism Phenotype items (the “with autistic people” and the “with non-autistic people” versions), separated by unrelated filler tasks. Therefore, the experiment was primarily a 2 (participant identity: autistic versus non-autistic) by 2 (item context: “with autistic people” versus “with non-autistic people”) mixed design.

### Methods

#### Materials: Broad autism phenotype questionnaire items

The experimental stimuli comprised all items on the Broad Autism Phenotype Questionnaire that involve social interaction or communication, which were all 12 items from the social interaction subscale (what the authors call the “Aloof” subscale), all 12 items from communication subscale (what the authors call the “Pragmatic Language” subscale), and 2 items from the personality subscale (what the authors call the “Rigid” subscale). Table 1 provides a list of the Experiment 1 stimuli, along with a list of the Broad Autism Phenotype items not included in Experiment 1.

**Table 1:** Experiment 1 Stimuli (Broad Autism Phenotype Questionnaire).

**Social Interaction Subscale**
I like being around [autistic/non-autistic] people.*
I prefer to be alone rather than with [autistic/non-autistic] people.
I enjoy being in social situations with [autistic/non-autistic] people.*
I feel like I really connect with [autistic/non-autistic] people.*
I look forward to situations where I can meet new [autistic/non-autistic] people.*
[Autistic/Non-autistic] acquaintances find it easy to approach me for casual (informal) interaction.*
I think that I am warm and friendly in my casual (informal) interactions with [autistic/non-autistic] acquaintances.*
I think that I am good at making small talk during casual (informal) interactions with [autistic/non-autistic] acquaintances.*
During casual (informal) interaction, I enjoy chatting with [autistic/non-autistic] acquaintances.*
When I make casual (informal) conversation with [autistic/non-autistic] acquaintances it is just to be polite.
Casual (informal) conversation with [autistic/non-autistic] acquaintances bores me.
I would rather talk to [autistic/non-autistic] people for the purpose of getting information than for the purpose of socializing.
**Communication Subscale**
In conversation with [autistic/non-autistic] people, it’s hard for me to avoid getting sidetracked (distracted by something that is not the main topic).
During casual (informal) conversations with [autistic/non-autistic] acquaintances, I feel disconnected or “out of sync.”
During casual (informal) conversations with [autistic/non-autistic] acquaintances, I feel “in-tune” with them.*
During casual (informal) conversations with [autistic/non-autistic] acquaintances, I can tell when someone is not interested in what I am saying.*
In casual (informal) conversation with [autistic/non-autistic] acquaintances, I can tell when it is time to change topics.*
I find it hard to get my words out smoothly when talking with [autistic/non-autistic] people.
I have been told by [autistic/non-autistic] people that I talk too much about certain topics.
I lose track of my original point when talking to [autistic/non-autistic] people.
I think my voice has a flat or monotone sound to it in conversation with [autistic/non-autistic] people.
I think that I leave long pauses in conversation with [autistic/non-autistic] people.
I think that I speak too loudly or softly [when I talk with [autistic/non-autistic] people.
[Autistic/Non-autistic] people ask me to repeat things I’ve said because they don’t understand.
**Personality Subscale**
[Autistic/Non-autistic] people get frustrated by my unwillingness to bend (compromise).
[Autistic/Non-autistic] people have to talk me into trying something new.
**Broad Autism Phenotype Questionnaire Items Not Used**
I feel a strong need for sameness from day to day.
I have a hard time dealing with changes in my routine.
I act very set in my ways.
I like to closely follow a routine while working.
I have to warm myself up to the idea of visiting an unfamiliar place.
I keep doing things the way I know, even if another way might be better.
I am comfortable with unexpected changes in plans.*
I am flexible about how things should be done.*
I look forward to trying new things.*
I alter my daily routine by trying something different.*

* Indicates that item is reverse scored.

Two material sets were formed from these stimuli. In the “with autistic people” material set, “people” were specified as “autistic people”; for example, the item “I like being around other people” appeared as “I like being around autistic people.” In the “with non-autistic people” material set, “people” were specified as “non-autistic people”; for example, the item “I like being around other people” appeared as “I like being around non-autistic people.” For items that only implicitly reference people in their original version, they explicitly referenced “people” in their specified versions. For example, “I enjoy being in social situations” was specified as “I enjoy being in social situations with autistic people” (in the “with autistic people” material set), and “I leave long pauses in conversation” was specified as “I think that I leave long pauses in conversation with non-autistic people” (in the “with non-autistic people” material set). The item “I think that I leave pauses in conversation” also illustrates the inclusion of “I think” as a preface, which was added to items that reference traits for which other people are often the judge (e.g., “I think my voice has a flat or monotone sound to it”). Experiment 1 specified the reference to be the respondents themselves to obviate any confusion. Experiment 2 examined this issue more directly.

The authors of the Broad Autism Phenotype Questionnaire annotated 10 of their original items with a footnote informing respondents that the social interaction or communication referenced in these items should be interpreted as occurring with “casual interaction with acquaintances, rather than special relationships such as with close friends and family members” ([21], p. 1689). Experiment 1 specified this interpretation in the items themselves. For example, the original item “I am good at making small talk” was specified as “I think that I am good at making small talk during casual (informal) interactions with [autistic/non-autistic] acquaintances.”

Whenever the original Broad Autism Phenotype item contained an idiom, Experiment 1 defined the idiom parenthetically (e.g., “sidetracked” is defined as “distracted by something that is not the main topic”). Clarifying vocabulary improves the validity of personality and behavior questionnaires [41] and is recommended as best practice [4]. Each item was accompanied by the six response choices typically used in the Broad Autism Phenotype Questionnaire: “Very often,” “Often,” “Somewhat often,” “Occasionally,” “Rarely,” and “Very rarely.” In addition to these six responses, participants in our study were given the response “Do not wish to say or not applicable.” This alternate response is often used to offset the requirement to respond to all items on Internet-based surveys [42,43].

The items appeared in each of the two material sets (the “with autistic people” material set and the “with non-autistic people” material set) in the same order as they appear on the Broad Autism Phenotype Questionnaire. At the beginning of the “with autistic people” material set, participants read the following instructions:

*In this section, there will be 26 items that ask about some of your personality traits and your social interactions and communication with autistic people. By autistic people, we mean people who identify as being on the autistic spectrum either because they have been formally diagnosed or because they have recognized that the criteria fit them*.

At the beginning of the “with non-autistic people” material set, participants read the following instructions:

*In this section, there will be 26 items that ask about some of your personality traits and your social interactions and communication with non-autistic people. By non-autistic people, we mean people who do not identify as being on the autistic spectrum, either because they have not been formally diagnosed or because the diagnostic criteria do not fit them*.

The instructions for both the “with autistic people” and “with non-autistic people” material sets also stated the following:

*If you are not sure how to respond to any of the 26 items in this section, just respond in the way that makes the most sense to you and then continue to the next item. If you would prefer not to respond to a particular item, please select “Do not wish to say or not applicable*.*”*

Half the participants were randomly assigned to complete the “with autistic people” material set first, followed by 10 minutes of filler tasks, and then they completed the “with non-autistic people” material set. The other half of the participants completed the “with non-autistic people” material set first, followed by 10 minutes of filler tasks, and then they completed the “with autistic people” material set. The filler tasks, described below, were unrelated to the experiment.

#### Materials: Filler tasks

As a buffer between completing the two material sets, participants completed three filler tasks. The three filler tasks were perceptual tests from the Perceptual Speed factor of Educational Testing Service’s kit of factor referenced tests [44]. The three tasks were Identical Pictures, Number Comparison, and Finding As. On each trial in Identical Pictures, participants were shown a target line-drawing, and their task was to identify that target line-drawing from a set of five line-drawings, which comprised the target line-drawing and four lures. On each trial in Number Comparison, participants were shown a pair of number sequences (e.g., 49471307 and 47471307), and their task was to identify whether the members of each pair were identical. For both Identical Pictures and Number Comparison, participants were given 90 seconds to complete as many trials as they could; they took a short break, and they were given another 90 seconds to complete as many more trials as they could.

On each trial in Finding As, participants were shown a list of approximately 40 words, and their task was to find the five words in that list that contained the letter a (e.g., ladder, instead, readily). Participants were given 120 seconds to complete as many lists as they could; they took a short break, and they were given another 120 seconds to complete as many more lists as they could. For all three filler tasks, participants were told “to work as quickly as you can without making too many errors” and “that it is not expected that you will finish all the items in the time allowed.”

#### Materials: Autistic group identity and contact

We administered a 20-item instrument to assess autistic group identity, exposure, and experience. The items were modified from instruments used to measure cultural identity, exposure, and experience and are available at www.GernsbacherLab.org. Because self-identification items appear on several cultural identity instruments (e.g., [45,46]), on the first item of our instrument, participants indicated whether they identify as an autistic person. Participants responded via three choices: “Yes,” “No,” and “Do not wish to say or not applicable.” On the next seven items, participants indicated whether their mother; father; partner or spouse; sibling; offspring; cousin; and nephew, niece, aunt, or uncle identify as an autistic person. Family identification items appear on several cultural contact instruments (e.g., [45,46]). For these seven items, participants responded via four choices: “Yes,” “No,” “Don’t know,” and “Do not wish to say or not applicable.”

On the next six items, participants indicated how many of their friends and colleagues are autistic. These six items (and the cultural contact instrument after which they were modeled) assayed participants’ current closest personal friends [47]; current closest coworkers, colleagues, or classmates [48]; top role models [49]; childhood friends, from age 5 to 12 [45]; childhood friends, from age 13 to 17 [45]; and adult friends, from age 18 and older [45]. For these six items, on which participants indicated how many of their friends and colleagues are autistic, participants responded via seven choices: “All or almost all are autistic,” “Most are autistic, but some are not autistic,” “About half are autistic, and half are not autistic,” “Most are not autistic, but some are autistic,” “All or almost all are not autistic,” “Don’t know,” and “Do not wish to say or not applicable.”

On the last six items, participants indicated how much of their social interaction and communication is with autistic persons. Two items assayed in-person social interaction (“I prefer attending social gatherings at which ….” modeled after [48], and “I would prefer to live in a community of people in which …” modeled after [50]), and one item assayed reading materials (“Of the blogs and websites I read …” modeled after [49]). The remaining three items assayed Internet-based interaction and communication, which are too new to be well represented in previous measures of cultural identity, exposure, and experience.

The three Internet-based communication and interaction items assayed synchronous online communication such as chat, instant messaging, and Skype; asynchronous online communication, such as email, listserves, Internet forums, and Internet discussion boards; and online social networking sites such as Facebook and YouTube. For these items, participants responded via seven choices: “All or almost all of the people I communicate with are autistic,” “Most of the people I communicate with are autistic, but some are not autistic,” “About half of the people I communicate with are autistic, and half are not autistic,” “Most of the people I communicate with are not autistic, but some are autistic,” “All or almost all of the people I communicate with are not autistic,” “Don’t know,” and “Do not wish to say or not applicable.”

Prior to completing the 20 group identity and contact items, participants read the following instructions:

*The 20 items in this section concern your experience interacting with autistic people. By autistic people, we mean people who identify as being on the autistic spectrum either because they have been formally diagnosed or because they have recognized that the criteria fit them. If you are not sure how to respond to any of the 20 items in this section, just respond in the way that makes the most sense to you and then continue to the next item. If you would prefer not to respond to a particular item, please select "Do not wish to say or not applicable*.*"*

#### Participants

For both experiments reported here, participants were recruited through the Gateway Project (http://thegatewayproject.org), which is an Internet-based research platform for inclusive, respectful, accessible, and relevant studies involving autistic and non-autistic adults. Participants in the Gateway Project first complete the Gateway Survey, which is a 30-minute questionnaire that collects demographic data, such as age, education, and gender and includes the 50-item Autism-Spectrum Quotient [20].

For both experiments reported here, autistic participants were defined as adults who met criteria for the autism spectrum on the Autism-Spectrum Quotient (score 31 or higher, i.e., agree with 62% or more of the Autism-Spectrum Quotient items) and who identified as autistic. Non-autistic participants were adults who did not meet criteria for the autism spectrum on the Autism-Spectrum Quotient (score 30 or lower, i.e., agree with 60% or fewer of the Autism-Spectrum Quotient items) and who did not identify as either being autistic or as having any other disability.

We computed the participants’ score on the Autism-Spectrum Quotient as a percentage because, in addition to the standard Autism-Spectrum Quotient response choices “Definitely agree,” “Slightly agree,” “Slightly disagree,” “Definitely disagree,” we offered participants the response choice “Do not wish to say.” Participants’ Autism-Spectrum Quotient scores were based on the items to which they responded other than “Do not wish to say,” and we required that participants respond to at least 85% of the Autism-Spectrum Quotient items with responses other than “Do not wish to say” to be included in the experiment.

We operationalized identifying as autistic or non-autistic via participants’ responses to the statement “I consider myself to be on the autistic spectrum (including Autistic Disorder, Asperger’s Disorder, and PDD-NOS).” The response choices were “Yes, and I have been formally diagnosed,” “Yes, but I have not been formally diagnosed,” “No,” and “Do not wish to say.” Autistic participants were those who responded with either of the two “Yes” choices, and non-autistic participants were those who responded “No.” Although we did not require autistic participants to have a formal autism diagnosis, the majority did, and the results of both experiments reported here replicated when we restricted our sample of autistic participants to only those with a formal diagnosis.

In Experiment 1, data were analyzed from 124 autistic and 124 non-autistic participants who were matched on age, sex, gender, and parental education. The participants’ characteristics are summarized in Table 2. Half the 124 autistic participants were randomly assigned to complete the “with autistic people” material set first, followed by 10 minutes of filler tasks, and then they completed the “with non-autistic people” material set. The other half of the 124 autistic participants completed the “with non-autistic people” material set first, followed by 10 minutes of filler tasks, and then they completed the “with autistic people” material set. The same was true for each half of the 124 non-autistic participants.

**Table 2:** Experiment 1 Participants’ Characteristics.

	Autistic Participants	Non-Autistic Participants	Test	
	*N* = 124	*N* = 124	Statistic	*p*
Autism-Spectrum Quotient (in percent): *M* (*SD*)	80.37 (8.939)	30.97 (12.28)	*t*(246) = 36.22	< .001
Formal Diagnosis: Yes/No	90/34	0/124	χ^2^(1) = 141.3	< .001
Age (in years): *M* (*SD*)	38.84 (12.79)	38.42 (12.28)	*t*(246) = 0.263	.793
Parent Education (in years): *M* (*SD*)	15.63 (2.751)	15.68 (2.704)	*t*(246) = -0.140	.889
Sex: Male/Female	62/62	62/62	χ^2^(1) = 0.000	1.000
Gender: Men/Women/Outside Gender Binary	61/60/3	62/62/0	χ^2^(2) = 3.041	.219
Latino or Hispanic: No/Yes	117/6^a^	120/4	χ^2^(1) = 0.434	.510
Racial Identity: White/Person of Color ^1^	112/11^a^	105/18^a^	χ^2^(5) = 6.826	.234
Country: USA/Other	94/30	115/9	χ^2^(1) = 13.42	< .001
Number of Autistic Relatives: *M (SD)* ^2^	1.342 (1.325)^d^	0.372 (0.672)^c^	*t*(176) = 7.155	< .001
Extent of Autistic Friends/Colleagues and				
Autistic Socializing/Communicating *M (SD)* ^3^	1.297 (0.777)^a^	0.267 (0.323)^b^	*t*(163) = 13.57	< .001

^a, b, c, d^ One, two, three, or four participants (respectively) did not want to respond to these items.

^1^ The six Racial Identity categories are American Indian or Alaska Native, Asian, Native Hawaiian or Other Pacific Islander, Black or African American, White, Multi-racial.

^2^ The number of autistic relatives is the sum of participants’ “Yes” responses to seven items on which participants indicate whether their (1) mother, (2) father, (3) partner or spouse, (4) sibling, (5) offspring, (6) cousin, and (7) nephew, niece, aunt, or uncle identify as an autistic person.

^3^ The extent of autistic friends/colleagues and autistic socializing/communicating is the average of participants’ responses to 12 items scored 0 (e.g., “All or almost all of the people I communicate with are not autistic”) to 4 (e.g., “All or almost all of the people I communicate with are autistic”).

For both experiments, the following checks ensured participant fidelity [51]. A) Participants must have recorded the same birthdate (in month and year) during the experiment as they recorded when they completed the Gateway Survey. B) Participants must have self-identified as autistic or non-autistic during the experiment in the same way as they self-identified during the Gateway Survey. C) Participants must have reported completing both the experiment and the Gateway Survey to the best of their ability (i.e., responded “Strongly agree,” “Somewhat agree,” or “Slightly agree” rather than “Do not agree” or “Do not wish to say” to the item “I completed this study to the best of my ability”). Seriousness checks are considered best practice in online studies [52].

For each of the two experiments reported here, participants were remunerated by being entered into a drawing with a 1 in 10 chance of winning a $25 Amazon gift certificate. Participants in the Gateway Project are allowed to participate in more than one of the Gateway studies. Of the 412 participants in Experiments 1 and 2, 169 participated in only Experiment 1; 85 participated in only Experiment 2; and 79 participated in both Experiments 1 and 2.

Participants were kept naïve about the research hypothesis. Experiment 1 was titled “Interaction Study A,” and the two material sets were titled “Your Personality Traits and Your Interactions with Autistic People” and “Your Personality Traits and Your Interactions with Non-Autistic People.” The term Broad Autism Phenotype Questionnaire was never mentioned. The filler tasks were described as “activities that you will perform next [that] are not associated with the items you just answered about your personality traits, social interactions, and communication.”

The autistic and non-autistic participants did not differ in their performance on the filler tasks: Identical Pictures (autistic participants: *M* = 25.69, *SD* = 7.460; non-autistic participants: *M* = 27.47, *SD* = 7.379; *t*(238) = 1.849, *p* = .066, *d* = -0.239); Number Comparison (autistic participants: *M* = 10.38, *SD* = 2.278; non-autistic participants: *M* = 10.82, *SD* = 2.822; *t*(238) = -1.340, *p* = .181, *d* = -0.173); Finding As (autistic participants: *M* = 20.92, *SD* = 7.664; non-autistic participants: *M* = 22.84, *SD* = 6.017; *t*(238) = -2.169, *p* = .031, *d* = -0.280).

#### Data analysis

Items on the Broad Autism Phenotype Questionnaire were scored in the standard way: 1 (“Very rarely”), 2 (“Rarely”), 3 (“Occasionally”), 4 (“Somewhat often”), 5 (“Often”), and 6 (“Very often”). Because participants were also allowed to respond to Broad Autism Phenotype items with the option “Do not wish to say or not applicable,” participants were required to respond to at least 85% of the items on both the “with autistic people” and the “with non-autistic people” material sets with responses other than “Do not wish to say or not applicable” to have their data included in the analyses.

The autistic and non-autistic participants did not differ in the percent of Broad Autism Phenotype items to which they responded other than “Do not wish to say or not applicable” (autistic participants: *M* = 98.57%, *SD* = 2.363%; non-autistic participants: *M* = 98.51%, *SD* = 2.523%; *F*(1,246) = 0.040, *p* = .842, η^2^_p_ = .000). However, both autistic and non-autistic participants responded less frequently with responses other than “Do not wish to say or not applicable” to Broad Autism Phenotype items on the “with autistic people” material set (*M* = 97.70%, *SD* = 4.107%) than on the “with non-autistic people” material set (*M* = 99.38%, *SD* = 1.856%, *F*(1,246) = 41.96, *p* < .001, η^2^_p_ = .146).

For the main analysis, participants’ responses to the Broad Autism Phenotype items were analyzed in a 2 (participant identity: autistic versus non-autistic, between-subjects) by 2 (item context: “with autistic people” versus “with non-autistic people,” within-subjects) by 2 (material set) mixed design Analysis of Variance (ANOVA), with planned comparisons conducted via *t*-tests. In both experiments reported here, a conservative α-level of .001 was used for all analyses.

#### Ethics statement

For both experiments reported here, participants provided written informed consent, and the experiments were approved by the Institutional Review Board at the University of Wisconsin–Madison (protocol SE-2008-0749 for the Gateway Survey, SE-2009-0187 for Experiment 1, and SE-2010-0441 for Experiment 2). In addition, the Gateway Council (a group of autistic and non-autistic researchers) ensured that the experiments were inclusive, respectful, accessible, and relevant.

### Results

**Fig 1:**
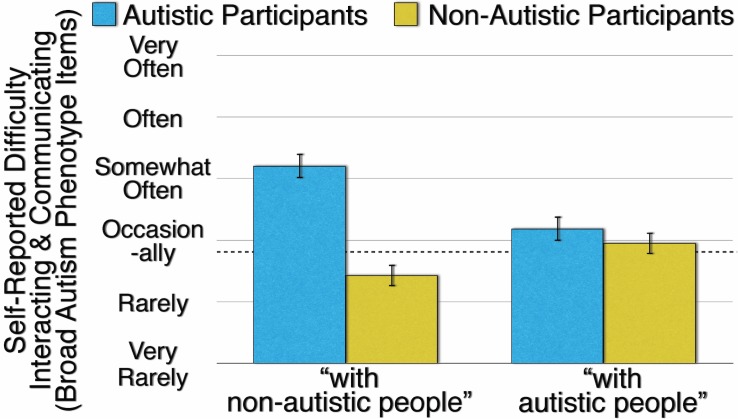
Autistic and Non-Autistic Participants’ Mean Self-Reported Difficulty Interacting and Communicating on Broad Autism Phenotype Items as a Function of Specifying the Interaction and Communication as “With Non-Autistic People” or “With Autistic People.” Error bars are 99.9% confidence intervals of the means. The dashed line indicates an impaired level of interaction and communication.

The results of Experiment 1 are presented in Fig 1. As predicted, autistic participants report significantly more difficulty interacting and communicating when the Broad Autism Phenotype Questionnaire items are specified as “with non-autistic people” (*M* = 4.205, *SD* = 0.658) than when the Broad Autism Phenotype items are specified as “with autistic people” (*M* = 3.185, *SD* = 0.682; *t*(123) = 13.13, *p* < .001, *d* = 1.180). In contrast, and as also predicted, non-autistic participants report significantly less difficulty interacting and communicating when the Broad Autism Phenotype items are specified as “with non-autistic people” (*M* = 2.430, *SD* = 0.596) than when the Broad Autism Phenotype items are specified as “with autistic people” (*M* = 2.952, *SD* = 0.596; *t*(123) = -9.189, *p* < .001, *d* = -0.825).

These two contrasting results, demonstrating an effect of in- versus out-group context on assessing autistic traits, produce a significant interaction (*F*(1,244) = 267.5, *p* < .001, η^2^_p_ = .523). The significant in-group/out-group interaction replicates when the sample of autistic participants is limited to those with a formal autism diagnosis (*F*(1,210) = 222.7, *p* < .001, η^2^_p_ = .515); when the Broad Autism Phenotype items, rather than the participants, are treated as random effects ([53,54]; *F*(1,25) = 89.46, *p* < .001, η^2^_p_ = .782); and when the female participants (*F*(1,120) = 230.4, *p* < .001, η^2^_p_ = .657) are analyzed separately from the male participants (*F*(1,120) = 78.58, *p* < .001, η^2^_p_ = .396).

The significant in-group/out-group interaction also replicates when the participants’ number of autistic relatives (*F*(1,236) = 182.5, *p* < .001, η^2^_p_ = .436) and the extent of the participants’ autistic friends, colleagues, socializing, and communicating (*F*(1,240) = 87.69, *p* < .001, η^2^_p_ = .268) serve as co-variates. The in-group/out-group interaction is unaffected by the order in which participants complete the two material sets (“with autistic people” first and “with non-autistic people” second or vice versa, *F*(1,244) = 0.513, *p* = .474, η^2^_p_ = .002). We feel confident asserting that the effect of in- versus out-group context on assessing autistic traits is robust.

Fig 1 illustrates, with a dashed line, the threshold that the Broad Autism Phenotype Questionnaire authors propose as indicating, for the items used in Experiment 1, an impaired level of social interaction and communication [21]. Autistic participants exceed that threshold, and non-autistic participants fall below that threshold, when the context for interacting and communicating is “with non-autistic people.” However, when the context is “with autistic people,” both autistic and non-autistic participants exceed the threshold; in fact, the autistic and non-autistic participants do not significantly differ from each other (*t*(246) = 2.865, *p* = .005, *d* = 0.364).

## Experiment 2

Experiment 2 examines the effect of reference group when assessing autistic traits. Experiment 2 manipulates three reference groups on the Social Responsiveness Scale: “According to autistic people,” “According to non-autistic people,” and “I think.” Half the participants in Experiment 2 identify as autistic, and half identify as non-autistic. Both autistic and non-autistic participants respond to the Social Responsiveness items using each of the three reference groups. Therefore, the experiment is primarily a 2 (participant identity: autistic versus non-autistic) by 3 (reference group: “According to autistic people” versus “According to non-autistic people” versus “I think”) mixed design.

### Methods

#### Materials: Social responsiveness scale items

The experimental stimuli comprised the 36 items on the Social Responsiveness Scale that assay traits observable both to other people and to oneself. For example, the items “I behave in ways that seem strange or bizarre,” “I have repetitive, odd behaviors,” and “I avoid eye contact or have unusual eye contact” assay traits that are observable to other people, as well as to oneself. Such items were included in the Experiment 2 stimuli. In contrast, the item “I seem much more uncomfortable in social situations than when alone” does not assay a trait that other people can observe (because other people cannot gauge how relatively uncomfortable a person is when they are alone). Similarly, the item “I am aware of what others are thinking or feeling” does not assay a trait that other people can observe (unless the other people are mind readers of mind reading). Although such items appear on the informant-report version of the Social Responsiveness Scale (e.g., “[my child] seems much more fidgety in social situations than when alone” and “[my spouse] is aware of what others are thinking or feeling”) they were not included in the Experiment 2 stimuli because they are not observable enough [55]. Thus, items such as “I can’t get my mind off something once I start thinking about it” were not included in Experiment 2, but items such as “I think or talk about the same thing over and over” were (because while repetitive thinking is not observable to others, repetitive talking is).

Experiment 2 also did not include the Social Responsiveness Scale item “I don’t recognize when others are trying to take advantage of me” because if a person is unable to recognize when others are taking advantage of them, they are unlikely to be able to report on that behavior (e.g., [56]). The decisions about inclusion of items were discussed among and confirmed by all co-authors, and 36 of the 65 items from the Social Responsiveness Scale were included in Experiment 2. Because the Social Responsiveness Scale is proprietary, we do not present a list of the items in this article. However, the list can be obtained by emailing the present article’s authors.

Two material sets were formed with these 36 items. In both material sets, all 36 items appeared with each of the three reference groups (“According to autistic people,” “According to non-autistic people,” and “I think”). The difference between the two material sets was the order in which the two external reference groups appeared. In one material set, each item appeared first with the reference group “According to non-autistic people,” then with the reference group “According to autistic people,” and then with the self-reference “I think.” In the other material set, each item appeared first with the reference group “According to autistic people,” then with the reference group “According to non-autistic people,” and then with the self-reference “I think.”

All idioms in the Social Responsiveness Scale items were defined, and each item was accompanied by the four response choices typically used in the Social Responsiveness Scale: “Almost always true,” “Often true,” “Sometimes true,” and “Not true.” In addition to these four choices, participants were given the two choices “Don’t know” and “Do not wish to say or not applicable.” The items appeared in each of the two material sets in the same order as they appear in the Social Responsiveness Scale, and participants read the following instructions:

*The following items concern how you think autistic people and non-autistic people view some of your traits, interests, habits, and social interactions. These items also concern how you view those things. By autistic people, we mean people who identify as being on the autistic spectrum either because they have been formally diagnosed or because they have recognized that the criteria fit them. By non-autistic people, we mean people who do not identify as being on the autistic spectrum either because they have not been formally diagnosed or because they recognize that the criteria do not fit them*.

*There are 36 sets of items in this section; each set has 3 items. These items are taken from a standardized measurement so that we can compare the results of this study with previous research. We apologize in advance if these items feel repetitive or are frustrating or offensive because that is not our intent*.

#### Materials: Autistic group identity and contact items

Experiment 2 used the same 20-item instrument to assess autistic group identity, exposure, and experience as Experiment 1.

#### Participants

Experiment 2 recruited and defined autistic and non-autistic participants the same way as Experiment 1. In Experiment 2, data were analyzed from 82 autistic and 82 non-autistic participants who were matched on age, sex, gender, and parental education. The participants’ demographic characteristics are summarized in Table 3. Thirty-eight of the 82 autistic participants and 38 of the 82 non-autistic participants were randomly assigned to the material set in which each item appeared first with the reference group “According to non-autistic people,” then with the reference group “According to autistic people,” and then with the self-reference “I think.” The other 44 autistic participants and 44 non-autistic participants were assigned to the material set in which each item appeared first with the reference group “According to autistic people,” then with the reference group “According to non-autistic people,” and then with the self-reference “I think.”

**Table 3:** Experiment 2 Participants’ Characteristics.

	Autistic Participants	Non-Autistic Participants	Test	
	*N* = 82	*N* = 82	Statistic	*p*
Autism-Spectrum Quotient (in percent): *M* (*SD*)	80.34 (9.229)	32.17 (12.14)	*t*(162) = 28.61	< .001
Formal Diagnosis: Yes/No	58/24	0/82	χ^2^(1) = 89.74	< .001
Age (in years): *M* (*SD*)	41.24 (12.59)	41.40 (12.43)	*t*(162) = -0.081	.935
Parent Education (in years): *M* (*SD*)	15.59 (2.712)	15.50 (2.686)	*t*(162) = 0.202	.840
Sex: Male/Female	41/41	41/41	χ^2^(1) = 0.000	1.000
Gender: Men/Women/Outside Gender Binary	41/38/3	41/41/0	χ^2^(2) = 3.114	.211
Latino or Hispanic: No/Yes	78/3^a^	80/2	χ^2^(1) = 0.219	.640
Racial Identity: White/Person of Color ^1^	75/4^b^	71/11	χ^2^(5) = 3.988	.551
Country: USA/Other	58/24	76/6	χ^2^(1) = 13.22	< .001
Number of Autistic Relatives: *M (SD)* ^2^	1.568 (1.360)^a^	0.383 (.663)^a^	*t*(116) = 7.052	< .001
Extent of Autistic Friends/Colleagues and				
Autistic Socializing/Communicating *M (SD)* ^3^	1.323 (0.810)	0.302 (0.391)^a^	*t*(117) = 10.26	< .001

^a, b^ One or three participants (respectively) did not want to respond to these items.

^1^ The six Racial Identity categories are American Indian or Alaska Native, Asian, Native Hawaiian or Other Pacific Islander, Black or African American, White, Multi-racial.

^2^ The number of autistic relatives is the sum of participants’ “Yes” responses to seven items on which participants indicate whether their (1) mother, (2) father, (3) partner or spouse, (4) sibling, (5) offspring, (6) cousin, and (7) nephew, niece, aunt, or uncle identify as an autistic person.

^3^ The extent of autistic friends/colleagues and autistic socializing/communicating is the average of participants’ responses to 12 items scored 0 (e.g., “All or almost all of the people I communicate with are not autistic”) to 4 (e.g., “All or almost all of the people I communicate with are autistic”).

Participants were kept naïve about the research hypothesis. The experiment was titled “Interaction Study B,” and the Social Responsiveness items were titled “Traits, Interests, Habits, and Social Interactions.” The term “Social Responsiveness Scale” was never mentioned.

#### Data analysis

Items on the Social Responsiveness Scale were scored in the standard way: 0 (“Not true”), 1 (“Somewhat true”), 2 (“Often true”), and 3 (“Almost always true”). Because participants in Experiment 2 were also allowed to respond to Social Responsiveness Scale items with the options “Do not know” and “Do not wish to say or not applicable,” to have their data analyzed, participants were required to respond to at least 85% of the Social Responsiveness items with the reference group “According to non-autistic people,” at least 85% of the items with the reference group “I think,” and at least two items with the reference group “According to autistic people” with responses other than “Do not know” or “Do not wish to say or not applicable.”

The autistic (*M* = 91.28%, *SD* = 9.835%) and non-autistic participants (*M* = 89.95%, *SD* = 10.72%) did not differ in the percent of Social Responsiveness items to which they responded other than “Do not know” or “Do not wish to say or not applicable” (*F*(1,162) = 0.688, *p* = .408, η^2^_p_ = .004). However, both the autistic and non-autistic participants responded less frequently with responses other than “Don’t know” or “Do not wish to say or not applicable” to Social Responsiveness items with the “According to autistic people” reference (*M* = 74.86%, *SD* = 29.39%) than with the “According to non-autistic people” (*M* = 98.32%, *SD* = 2.780%) or the “I think” reference (*M* = 98.66%, *SD* = 2.527%; *t*(163) = -10.43, *p* < .001, *d* = -0.815 and *t*(163) = -10.48, *p* < .001, *d* = -0.819, respectively).

For the main analysis, participants’ responses to the Social Responsiveness items were analyzed with a 2 (participant identity: autistic versus non-autistic, between-subjects) by 3 (reference group: “According to autistic people” versus “According to non-autistic people” versus “I think,” within-subjects) by 2 (material set) mixed design Analysis of Variance (ANOVA), with planned comparisons conducted via *t*-tests.

### Results

**Fig 2:**
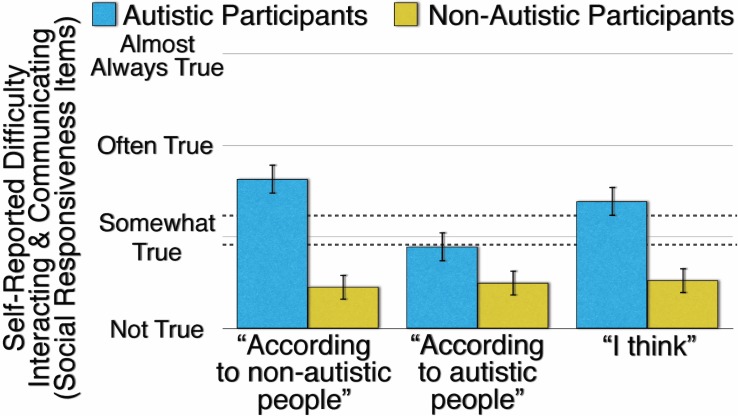
Autistic and Non-Autistic Participants’ Mean Self-Reported Difficulty Interacting and Communicating on the Social Responsiveness Scale Items as a Function of Reference Group (“According to Non-Autistic People,” “According to Autistic People,” and “I Think”). Error bars are 99.9% confidence intervals of the means. The higher dashed line indicates a severe level and the lower dashed line indicates a mild to moderate level of difficulty interacting and communicating.

The results of Experiment 2 are presented in Fig 2. As predicted, autistic participants’ difficulty interacting and communicating, as self-reported with the Social Responsiveness items, varies as a function of reference group (*F*(2,160) = 94.38, *p* < .001, η^2^_p_ = .541). With the out-group reference “According to non-autistic people” (*M* = 1.631, *SD* = 0.491) rather than “I think” (*M* = 1.388, *SD* = 0.351), autistic participants accentuate their difficulty interacting and communicating (*t*(81) = 5.222, *p* < .001, *d* = 0.577). In contrast, with the in-group reference “According to autistic people” (*M* = 0.893, *SD* = 0.456) rather than “I think,” autistic participants attenuate their difficulty interacting and communicating (*t*(81) = -10.15, *p* < .001, *d* = -1.121).

However, as Fig 2 also illustrates and contrary to predictions, non-autistic participants’ difficulty interacting and communicating, as self-reported with Social Responsiveness items, does not vary as a function of reference group (*F*(2,160) = 1.551, *p* = 0.215, η^2^_p_ = .019). Non-autistic participants report a similar level of difficulty interacting and communicating with the in-group reference “According to non-autistic people” (*M* = 0.454, *SD* = 0.315) as with the out-group reference “According to autistic people” (*M* = 0.499, *SD* = 0.494) and the self-reference “I think” (*M* = 0.525, *SD* = 0.360).

These two contrasting effects, the autistic participants’ sensitivity to reference group and the non-autistic participants’ insensitivity to reference group, produce a significant interaction (*F*(2,320) = 66.23, *p* < .001, η^2^_p_ = .293), although not the predicted interaction (which was that both autistic and non-autistic participants accentuate their stereotypical traits with an out-group reference and attenuate their stereotypical traits with an in-group reference). Only the autistic participants manifest the predicted effect of reference group; only the autistic participants appear sensitive to a reference group.

Autistic participants’ sensitivity to reference group replicates when the sample of autistic participants is limited to participants with a formal autism diagnosis (*F*(2,112) = 75.60, *p* < .001, η^2^_p_ = .574); when items are considered random effects rather than participants (*F*(2,70) = 146.2, *p* < .001, η^2^_p_ = .807); when only female autistic participants are considered (*F*(2,78) = 48.70, *p* < .001, η^2^_p_ = .555); when only male autistic participants are considered (*F*(2,78) = 43.58, *p* < .001, η^2^_p_ = .528); and when participants’ number of autistic relatives (*F*(2,156) = 51.14, *p* < .001, η^2^_p_ = .396) and the extent of their autistic friends, colleagues, socializing, and communicating (*F*(2,158) = 16.82, *p* < .001, η^2^_p_ = .176) serve as covariates.

Similarly, non-autistic participants’ insensitivity to reference group replicates when items are considered random effects (*F*(2,70) = 4.662, *p* = .013, η^2^_p_ = .118); when only female non-autistic participants are considered (*F*(2,78) = 1.106, *p* = .367, η^2^_p_ = .025); when only male non-autistic participants are considered (*F*(2,78) = 1.230, *p* = .298, η^2^_p_ = .031); and when participants’ number of autistic relatives (*F*(2,156) = 1.149, *p* = .320, η^2^_p_ = .015) and extent of autistic friends, colleagues, socializing, and communicating (*F*(2,156) = 1.046, *p* = .354, η^2^_p_ = .013) serve as covariates. Autistic participants’ sensitivity and non-autistic participants’ insensitivity to reference group are unaffected by whether participants respond first with the reference group “According to autistic people” or first with the reference group “According to non-autistic people” (*F*(2,160) = 1.076, *p* = .343, η^2^_p_ = .013; *F*(2,160) = 0.009, *p* = .991, η^2^_p_ = .000). Therefore, we feel confident asserting that, for the autistic participants, the effect of in- versus out-group reference on assessing autistic traits is robust.

Fig 2 illustrates, with dashed lines, two thresholds proposed by the Social Responsiveness Scales’ authors [22]. Scores above the lower threshold indicate “deficiencies … that are clinically significant and result in mild to moderate interference in everyday social interactions” and scores above the upper threshold indicate “a more severe interference in everyday social interactions” ([22], p. 657). As Fig 2 illustrates, autistic participants exceed both the “mild to moderate” and the “severe” threshold in their difficulty interacting and communicating with the out-group reference of “According to non-autistic people” and “I think.” In contrast, autistic participants’ difficulty interacting and communicating falls below the “mild to moderate” threshold with the in-group reference of “According to autistic people.” Non-autistic participants’ difficulty interacting and communicating never rises above the “mild to moderate” threshold, regardless of the reference group. In the General Discussion, we further consider why non-autistic participants appear insensitive to reference group.

## Exploratory analyses

We conducted exploratory analyses by taking advantage of the fact that when enrolling in our Gateway Project, participants complete the Autism-Spectrum Quotient without any specification of the items. Therefore, we could correlate participants’ responses to the unspecified Autism-Spectrum Quotient items with their responses to the context-specified Broad Autism Phenotype items from Experiment 1. We could also correlate participants’ responses to the unspecified Autism-Spectrum Quotient items with their responses to the reference-group-specified Social Responsiveness items from Experiment 2. Through these exploratory analyses, we can observe which context and which reference group autistic and non-autistic participants implicitly rely upon when the context and the reference group are not specified.

### Methods

#### Materials: Autism-Spectrum Quotient items

The administration and scoring of the Autism-Spectrum Quotient items were described in Experiment 1 (Participants subsection).

#### Data analysis

Because these analyses are exploratory, we refrained from null hypothesis testing. No α-level was established, and we interpret the correlation coefficients descriptively [57].

### Results

**Fig 3:**
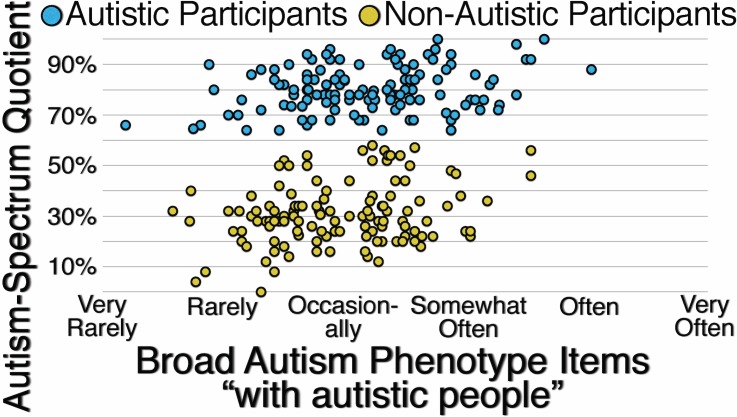
Correlation Between Autistic and Non-Autistic Participants’ Responses to Unspecified Autism-Spectrum Quotient Items and Their Responses to Broad Autism Phenotype Items Specified with the Context “With Autistic People.”

Fig 3 presents the correlations between participants’ responses to unspecified Autism-Spectrum Quotient items and participants’ responses to Broad Autism Phenotype items specified with the context of “with autistic people.” For autistic participants (*r*(122) = .250, *p* = .005), for non-autistic participants (*r*(122) = .264, *p* = .003), and for all participants (*r*(246) = .263, *p* < .001), the correlations are weak, suggesting that when the context for responding to items assessing autistic traits is not specified, neither autistic nor non-autistic participants are likely to use “with autistic people” as the implied context.

**Fig 4:**
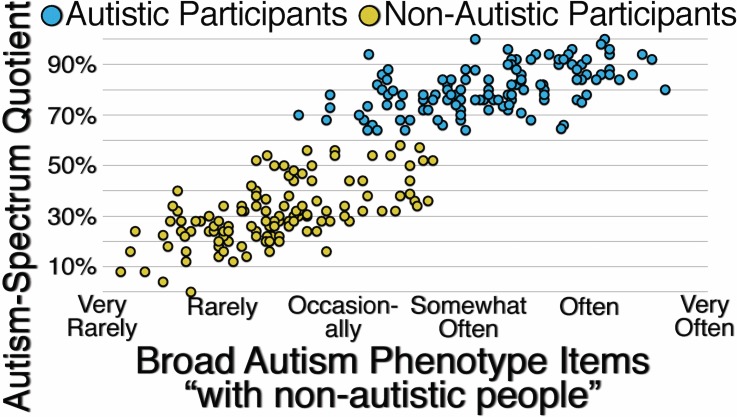
Correlation Between Autistic and Non-Autistic Participants’ Responses to Unspecified Autism-Spectrum Quotient Items and Their Responses to Broad Autism Phenotype Items Specified with the Context “With Non-Autistic People.”

Fig 4 presents the correlations between participants’ responses to unspecified Autism-Spectrum Quotient items and Broad Autism Phenotype items specified with the context of “with non-autistic people.” For autistic participants (*r*(122) = .519, *p* < .001) and for non-autistic participants (*r*(122) = .617, *p* < .001), the correlations are moderate to strong. For all participants (*r*(246) = .879, *p* < .001), the correlation is also strong, suggesting that when the context for responding to items assessing autistic traits is not specified, both autistic and non-autistic participants are likely to use “with non-autistic people” as the implied context.

**Fig 5:**
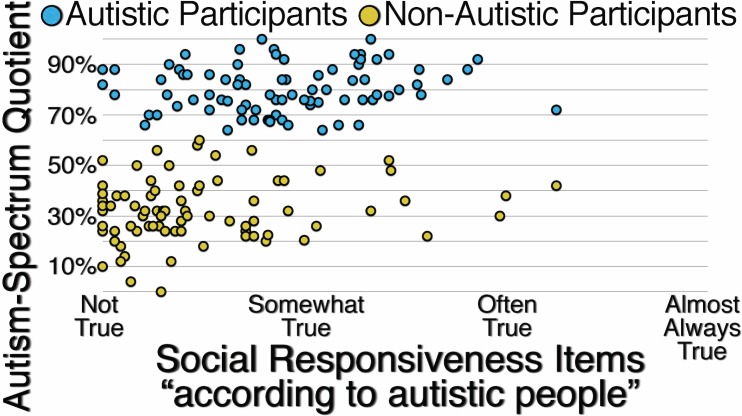
Correlation Between Autistic and Non-Autistic Participants’ Responses to Unspecified Autism-Spectrum Quotient Items and Their Responses to Social Responsiveness Items Specified with the Reference Group “According to Autistic People.”

Fig 5 presents the correlations between participants’ responses to unspecified Autism-Spectrum Quotient items and participants’ responses to Social Responsiveness items specified with the reference group “According to autistic people.” For autistic participants (*r*(80) = .113, *p* = .311), for non-autistic participants (*r*(80) = .180, *p* = .105), and for all participants (*r*(162) = .408, *p* < .001), the correlations are weak or moderate, suggesting that when the reference group for responding to items assessing autistic traits is not specified, neither autistic nor non-autistic participants are likely to use “According to autistic people” as the implied reference group.

**Fig 6:**
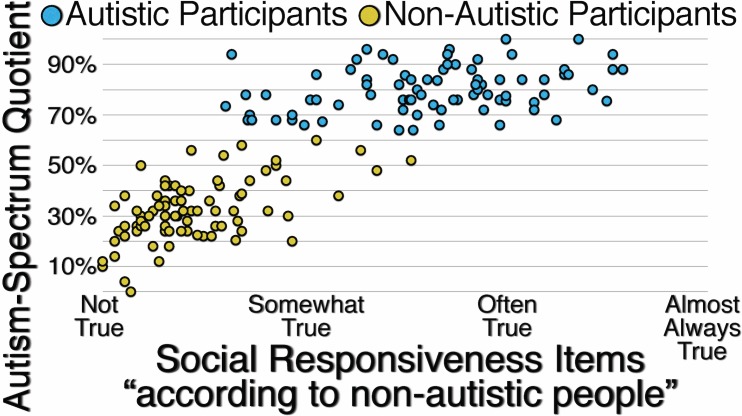
Correlation Between Autistic and Non-Autistic Participants’ Responses to Unspecified Autism-Spectrum Quotient Items and Their Responses to Social Responsiveness Items Specified with the Reference Group “According to Non-Autistic People.”

Fig 6 presents the correlations between participants’ responses to unspecified Autism-Spectrum Quotient items and Social Responsiveness items specified with the reference group “According to non-autistic people.” For autistic participants (*r*(80) = .319, *p* = .003), for non-autistic participants (*r*(80) = .562, *p* < .001), and for all participants (*r*(162) = .844, *p* < .001), the correlations are moderate to strong, suggesting that when the reference group for responding to items assessing autistic traits is not specified, both autistic and non-autistic participants are likely to use “According to non-autistic people” as the implied reference group.

**Fig 7:**
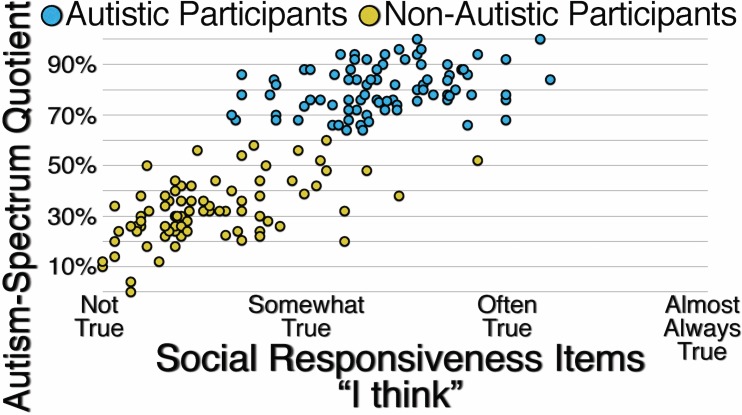
Correlation Between Autistic and Non-Autistic Participants’ Responses to Unspecified Autism-Spectrum Quotient Items and Their Responses to Social Responsiveness Items Specified with the Reference “I Think.”

Fig 7 presents the correlations between participants’ responses to unspecified Autism-Spectrum Quotient items and Social Responsiveness items specified with the reference “I think.” For autistic participants (*r*(80) = .292, *p* = .008) the correlation is weak, but for non-autistic participants (*r*(80) = .529, *p* < .001) and all participants (*r*(162) = .816, *p* < .001) the correlations are strong, suggesting that when the reference group for responding to items assessing autistic traits is not specified, non-autistic participants are likely to use “I think” as the implied reference.

As Table 4 illustrates, the pattern of these correlations remains the same when, instead of including all 50 items on the Autism-Spectrum Quotient, the analyses include only the 32 Autism-Spectrum Quotient items from the social interaction (ten items), communication (ten items), and attention switching subscales (ten items), and the two items from the attention to detail subscale that reference other people. As Table 4 also illustrates, the pattern of these correlations also remains the same when, instead of including all 50 items on the Autism-Spectrum Quotient, the analyses include only the 20 Autism-Spectrum Quotient items from the social interaction (ten items) and communication (ten items) subscales.

**Table 4:** Exploratory Correlations with Unspecified Autism-Spectrum Quotient Items.

	Broad Autism Phenotype Items “With Autistic People”		
	Autistic Participants	Non-Autistic Participants	All Participants
Autism-Spectrum Quotient (50)	*r*(122) = .250, *p* = .005	*r*(122) = .264, *p* = .003	*r*(246) = .263, *p* < .001
Autism-Spectrum Quotient (32)	*r*(122) = .230, *p* = .010	*r*(122) = .266, *p* = .003	*r*(246) = .259, *p* < .001
Autism-Spectrum Quotient (20)	*r*(122) = .237, *p* = .008	*r*(122) = .237, *p* = .008	*r*(246) = .259, *p* < .001
	Broad Autism Phenotype Items “With Non-Autistic People”		
	Autistic Participants	Non-Autistic Participants	All Participants
Autism-Spectrum Quotient (50)	*r*(122) = .519, *p* < .001	*r*(122) = .617, *p* < .001	*r*(246) = .879, *p* < .001
Autism-Spectrum Quotient (32)	*r*(122) = .531, *p* < .001	*r*(122) = .565, *p* < .001	*r*(246) = .869, *p* < .001
Autism-Spectrum Quotient (20)	*r*(122) = .510, *p* < .001	*r*(122) = .533, *p* < .001	*r*(246) = .859, *p* < .001
	Social Responsiveness Items “According to Autistic People”		
	Autistic Participants	Non-Autistic Participants	All Participants
Autism-Spectrum Quotient (50)	*r*(80) = .113, *p* = .311	*r*(80) = .180, *p* = .105	*r*(162) = .408, *p* < .001
Autism-Spectrum Quotient (32)	*r*(80) = .127, *p* = .256	*r*(80) = .136, *p* = .224	*r*(162) = .399, *p* < .001
Autism-Spectrum Quotient (20)	*r*(80) = .118, *p* = .290	*r*(80) = .104, *p* = .351	*r*(162) = .387, *p* < .001
	Social Responsiveness Items “According to Non-Autistic People”		
	Autistic Participants	Non-Autistic Participants	All Participants
Autism-Spectrum Quotient (50)	*r*(80) = .319, *p* = .003	*r*(80) = .562, *p* < .001	*r*(162) = .844, *p* < .001
Autism-Spectrum Quotient (32)	*r*(80) = .409, *p* < .001	*r*(80) = .565, *p* < .001	*r*(162) = .849, *p* < .001
Autism-Spectrum Quotient (20)	*r*(80) = .419, *p* < .001	*r*(80) = .530, *p* < .001	*r*(162) = .840, *p* < .001
	Social Responsiveness Items “I Think”		
	Autistic Participants	Non-Autistic Participants	All Participants
Autism-Spectrum Quotient (50)	*r*(80) = .292, *p* = .008	*r*(80) = .529, *p* < .001	*r*(162) = .816, *p* < .001
Autism-Spectrum Quotient (32)	*r*(80) = .305, *p* = .005	*r*(80) = .535, *p* < .001	*r*(162) = .818, *p* < .001
Autism-Spectrum Quotient (20)	*r*(80) = .280, *p* = .011	*r*(80) = .511, *p* < .001	*r*(162) = .807, *p* < .001

## General discussion

Because many of the personality and behavioral traits known to be sensitive to context and to reference groups (e.g., social imperviousness, directness in conversation, lack of imagination, affinity for solitude, difficulty displaying emotions) also appear in questionnaire-based assessments of autistic traits, these experiments investigate the effects of context and reference group on assessing autistic traits. The results demonstrate that specific contexts and specific reference groups matter when assessing autistic traits in autistic and non-autistic participants.

Experiment 1 demonstrates that specific contexts matter. When the context of the Broad Autism Phenotype Questionnaire is specified as the participants’ out-group (e.g., “I like being around non-autistic people” or “I like being around autistic people”), both autistic and non-autistic participants self-report having more autistic traits; when the context is specified as the participants’ in-group, both autistic and non-autistic participants report having fewer autistic traits.

Experiment 2 demonstrates that specific reference groups matter. When the reference group on the Social Responsiveness Scale is specified as the participants’ out-group (e.g., “According to non-autistic people, I have unusual eye contact”), autistic participants report having more autistic traits; when the reference group is specified as the participants’ in-group (e.g., “According to autistic people, I have unusual eye contact”), autistic participants report having fewer autistic traits. Non-autistic participants appear insensitive to reference group on the Social Responsiveness Scale, the reasons for which we discuss below.

Exploratory analyses suggest that when neither the context nor the reference group is specified (for assessing autistic traits on the Autism-Spectrum Quotient), both autistic and non-autistic participants are more likely to use the majority (“non-autistic people”) as the implied context and the implied reference group. Although these analyses are exploratory, their results are not surprising. The same pattern occurs when assessing other minority traits, which we discuss below.

### Non-autistic participants’ insensitivity to reference group

One possible reason why non-autistic participants appear insensitive to the reference group manipulation of Experiment 2 is that non-autistic participants might be insufficiently familiar with the out-group of autistic people to respond differentially to autistic people as a reference group. This reason seems unlikely because the non-autistic participants in Experiment 1 were sufficiently familiar with the out-group of autistic people to respond differentially to autistic people as a context effect. And the non-autistic participants in Experiment 2 are drawn from the same population as the non-autistic participants in Experiment 1.

Moreover, even if the non-autistic participants in Experiment 2 are unable to distinguish between how they are seen by autistic people and how they see themselves, they should nonetheless be able to distinguish between how they are seen by other non-autistic people and how they see themselves; that distinction is made quite readily by non-autistic people [58–60]. Indeed, the distinction between self- versus other-report can be quite striking when assessing non-autistic behavior [61] and personality [62,63], including social interaction [55] and communication [64]. Therefore, we do not think the reason why the non-autistic participants in Experiment 2 appear insensitive to reference group is due solely to their lack of familiarity with autistic people.

A second reason why the non-autistic participants in Experiment 2 appear insensitive to reference group may be the nature of the Social Responsiveness Scale. Perhaps the severe phrasing of so many of the Social Responsiveness items makes it difficult for non-autistic participants to respond with much variability. For example, in Experiment 2, few non-autistic participants, regardless of the reference group, report that they “react to people as if they are objects,” “behave in ways that seem strange or bizarre,” are “regarded by others as odd or weird,” “wander aimlessly from one activity to another,” have “repetitive, odd behaviors,” “touch or greet others in an unusual way,” “talk to people … like a robot,” “show … strange repetitive ways of handling or manipulating small items,” or are “too silly or laugh inappropriately.”

**Fig 8:**
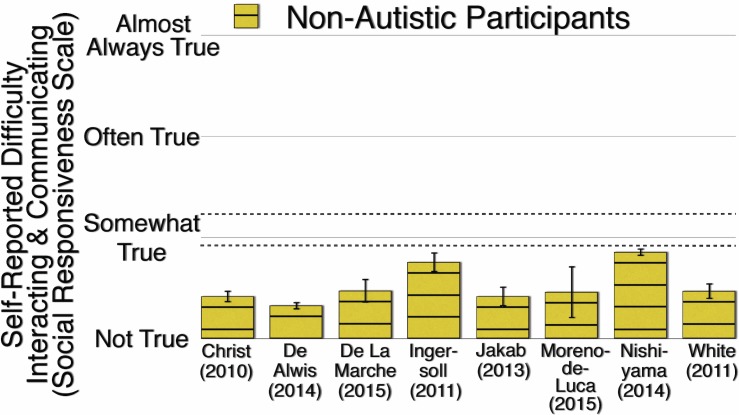
Non-Autistic Participants’ Mean Self-Reported Difficulty Interacting and Communicating on the Social Responsiveness Scale. Error bars are 99.9% confidence intervals of the means. The higher dashed line indicates a severe level and the lower dashed line indicates a mild to moderate level of difficulty interacting and communicating.

Therefore, non-autistic participants’ responses to Social Responsiveness items might be too bound to the scale’s floor to show any variability of reference group. In other studies, when non-autistic participants respond to Social Responsiveness items, they too show floor-like levels of self-reported difficulty interacting and communicating. Fig 8 illustrates such data from eight other studies, many with large samples of non-autistic participants ([65], *N* = 1847; [66], *N* = 3080; [67], *N* = 301; [34], *N* = 601; [35], *N* = 127; [68], *N* = 51; [24], *N* = 3147; [69], *N* = 667). The mean of the means illustrated in Fig 8 (0.523) is similar to the mean of the non-autistic participants in Experiment 2 (0.525 for “I think”).

Other researchers also note that “a weakness of the [Social Responsiveness Scale] is that it is not normally distributed” ([70], p. 626); non-autistic participants’ self-reports skew sharply to the bottom of the scale. For example, in one study, the majority of the non-autistic participants self-report “Never true” to almost 90% of the items, and one out of five participants self-report “Never true” to every item [66]. In our study, the majority of non-autistic participants also self-report “Not true” to the majority of the Social Responsiveness items (when specified with the reference “I think”). Therefore, it is possible that the non-autistic participants in our study seem insensitive to reference group because the severe phrasing of the Social Responsiveness items makes it difficult to capture a non-autistic range of self-reported difficulty interacting and communicating (despite the fact that the Social Responsiveness Scale is intended to be administered to non-autistic persons as a way to identify the degree of their “sub-threshold” autistic traits [22]).

### Majority versus minority context and reference groups

Our exploratory analyses suggest that when neither the context nor the reference group is specified for assessing autistic traits, both autistic and non-autistic participants use the majority as the implied context and reference group. We are not surprised by this result. The positions and perspectives held by the majority are usually more valued than those held by the minority [71], even for members of the minority [72]. Assessments of racial minority group members’ behavior used to be based implicitly on the context and reference group of racial majority group members [73], even when those assessments were made by members of racial minority groups [74]. Assessments of gay and lesbian behavior are often still based implicitly on the context and reference group of heterosexual behavior [75].

Assessments of women’s behavior can even be based implicitly on the context and reference group of men’s behavior [76], suggesting that implicit contexts and reference groups are not driven by numerical majority but are instead driven by status [77]. Correspondingly, the groups that form implicit contexts and reference groups are projected to have higher status [78]. Therefore, it is unsurprising that assessment of autistic (minority and lower status) traits would be based implicitly on the context and reference group of non-autistic (majority and higher status) people.

### Self- versus other-report of autistic traits

The experiments reported here demonstrate that, contrary to some assertions [25], autistic participants are adept at self-reporting their autistic traits. Indeed, these experiments demonstrate that autistic participants might be even more adept than non-autistic participants at self-reporting their autistic traits, as illustrated by the autistic participants’ greater sensitivity to reference group in Experiment 2. Perhaps autistic participants are more adept at self-reporting their traits because of their greater “internal focus of attention,” which is “one of the simplest factors … related to accurate self-assessment” ([79], p. 517).

Some of the assertions that autistic participants are unequipped to self-report their traits are driven by the popular assumption that autistic people lack a ‘theory of mind’ [80]. Assumedly, autistic people lack the understanding that they have a mind, much less that other people have a mind. The data presented here join other bodies of empirical evidence that argue against the popular, but empirically weak, assumption that autistic people lack a theory of mind [81,82]. The data presented here demonstrate that autistic participants are well equipped not only to self-report on their own traits, but also to self-report on their traits in different contexts and to self-report on how others view their traits.

Other assertions that autistic participants are less skilled at self-reporting their traits are driven by studies reporting a discrepancy between parents’ report of their autistic offspring’s traits and those offspring’s own self-report. However, when objective data are available to adjudicate the discrepancy, autistic offspring’s self-reports, rather than their parents’ reports, align closer to the objective data (e.g., [83,84]. Compared with objective assessments, parents under-estimate their autistic offspring’s intelligence [85], they over-estimate their offspring’s anxiety [83], and they poorly estimate their offspring’s autistic traits [86–91]. Therefore, most likely it is parents’ assessments of their autistic offspring’s traits, rather than autistic offspring’s self-report of their own traits, that are not well calibrated.

Discrepancies between parent-report and offspring self-report are well established in the general literature [92–94]. Discrepancies between parent-report and offspring self-report are also well established in the disability literature for parents of offspring with a variety of disabilities other than autism (intellectual disability, [95]; juvenile arthritis, [96]; Duchenne muscular dystrophy, [97]; visual impairment, [98]; cerebral palsy, [99]). Similarly, persons with disabilities other than autism have also been assumed to be unequipped to self-report on their traits; for example, persons with physical disabilities have been assumed to be less skilled at self-reporting their difficulty interacting and communicating with non-disabled people [100,101]. However, as is the case with parent-report and autistic offspring’s self-report, when objective data are available to adjudicate the discrepancy between parent-report and otherwise disabled offspring’s self-report, disabled offspring’s self-report aligns closer to the objective data than their parents’ report do (e.g., [102]).

Factors that are known to bias parents’ report of their non-autistic offspring’s traits [103,104] also bias parents’ report of their autistic offspring’s traits. These factors include parents’ implicit comparisons with their other offspring [105] and the parents’ own mental health [106]. For example, parents’ report of their offspring’s autistic traits (on the Social Responsiveness Scale) is better predicted by those parents’ self-report of their own depression than by objective measures of their offspring’s autistic traits [107].

Factors that are known to increase the accuracy of parent-report and other proxy-report [61–63,108] also increase the accuracy of parents’ report of their offspring’s autistic traits. These factors include the nature of the assessment [109] and the observability of the traits that parents are assessing [110–112]. As White et al. ([113], p. 50) note, many parent-report items “require subjective inference about the child’s inner experiences … Unless the child has verbalized the specific experience to the parent, parents are left to infer … leading to inherent imprecision of measurement.”

We agree with Warren et al. [114] who advocate for improving parents’ accuracy in reporting their offspring’s autistic traits. Warren et al. [114] recommend incorporating into parents’ assessment of autistic traits validity techniques that are commonly used when assessing other personality and behavioral traits (e.g., including low-frequency items to detect over-endorsement and applying statistical analysis to identify inconsistent endorsement). Although the goal of our research has been to examine social psychological rather than psychometric factors that affect self-assessment of autistic traits, we extend Warren et al.’s [114] psychometric recommendations to the assessment of autistic traits via self-report.

### Conclusions

We conclude by offering two caveats concerning the assessment of autistic traits. First, our data illustrate that it is important to specify the context (with whom?) and the reference group (according to whom?) when assessing autistic traits. If either the context or the reference group is left unspecified, respondents are likely to report their difficulty interacting and communicating in the context of non-autistic people and with the reference group of non-autistic people. Therefore, if a non-autistic context or reference group is not intended, the assessment instrument should be further specified.

Second, our data illustrate that both autistic and non-autistic people’s difficulty interacting and communicating is contextually specific. Both groups can more easily interact and communicate with their in-group (i.e., people similar to themselves) than with their out-group (i.e., people dissimilar to themselves). This finding, although consistent with social psychological principles, bears implications not only for accurately assessing autistic traits but also for designing optimal environments that enable successful interaction and communication.
